# Chinese physical fitness standard for campus football players: A pilot study of 765 children aged 9 to 11

**DOI:** 10.3389/fphys.2022.1023910

**Published:** 2022-10-05

**Authors:** Hong Jia, Bin Wan, Te Bu, Yang Luo, Weiping Ma, Sen Huang, Liang Gang, Wei Deng, Zeyong Liu

**Affiliations:** ^1^ College of Physical Education, Hunan Normal University, Changsha, China; ^2^ Hunan Kuying Sports Development Co Ltd, Changsha, China; ^3^ Hunan Institute of Sport Science, Changsha, China; ^4^ Hunan Biological Electromechanical Vocational Technical College, Changsha, China; ^5^ School of Foreign Language and Culture, Guangdong University of Finance, Guangzhou, China

**Keywords:** GAMLSS, big data, flexibility, agility, speed, power, soccer training

## Abstract

**Objectives:** In 2022, 55 million Chinese children participate in campus football; however, there is no physical fitness standard, making it a priority task to enhance the current national program. This study aimed to explore a pilot method for the development of a reliable physical fitness standard.

**Methods:** This study examined 765 male football players aged 9 to 11 in 2020 and 2022. The anthropometric and physical fitness assessments were conducted in accordance with the Chinese Football Association’s field manuel. Physical fitness tests include sit and reach test, *t* test, 30 m run test, and vertical jump test. Physical fitness standard was modeled using the generalized additive models for location, scale, and shape (GAMLSS). Data were fitted with appropriate GAMLSS distributions and smoothing term. P-splines were applied to smooth the model’s parameters using the default local maximum likelihood method and link functions. Following diagnostics of fitted models, age-specific centile estimations were computed for physical fitness tests. In addition, players in each age group were categorized according to their body mass index as normal weight or overweight/obese. Welch’s *t-*test was utilized to compare the group differences in physical fitness testing. The significance level was chosen at *p* < 0.05.

**Results:** Sit and reach test, *t* test, 30 m run test, and vertical jump test data were fitted with original Sinh-Arcsinh, Box-Cox power exponential, Box-Cox power exponential, and Box-Cox Cole and Green, respectively. Physical fitness standard for each age group is presented as tabulated centiles (1p, 3p, 5p, 15p, 25p, 50p, 75p, 85p, 95p, 97p, 99p). Overweight/obese campus football players did significantly worse (*p* < 0.05) on the *t* test, 30 m run test, and vertical jump test than their normal-weight peers of the same age.

**Conclusion:** This study developed the first physical fitness standard for 9 to 11-year-old campus football players in China. We made three recommendations to Chinese policymakers on sample size, data management, and field procedure for the creation of a national physical fitness standard.

## Introduction

In 2015, the General Office of the State Council of the People’s Republic of China issued the “Comprehensive Plan for the Reform and Development of Chinese Football” (The State Council of the People’s Republic of China, 2015), which stated that reforming and promoting the development of national youth campus football (hereafter referred to as campus football) is essential for promoting the popularization of football (soccer) education, facilitating the joint development of academic learning and physical fitness, and promoting the rapid growth of youth football talent. Since its inception, the Ministry of Education has coordinated this national program through four initiatives to promote the development of campus football, including the construction of schools with campus football characteristics, the establishment of a youth competition system, the establishment of “all-star” training camps, and the establishment of an honor incentive system. In 2022, there are about 30,000 schools with campus football characteristics, and 55 million children play in football (Liu, 2022), making it the largest organized campus sport in China.

Meanwhile, as campus football expands rapidly, new challenges continue to surface, necessitating ongoing scientific optimization of the program. For example, our group has observed that injury prevention and management strategies lag far behind the mainstream national development of campus football (Liu et al., 2022), which, in the long term, could hinder the healthy development and growth of children. The scientific evaluation of player development represents another significant deficit. The State Council explicitly stated in the action plan (The State Council of the People’s Republic of China, 2020) that it is necessary to “develop a rating assessment and evaluation method”, “continual improvement of the campus football and youth training system utilizing the same age group tournament selection method”, and “further strengthen the policy on the advancement of excellent campus football players in each school section”. In practice, there are no scientifically defined objective athletic evaluation methods, age-specific player selection methods, or “excellent” college admission criteria.

To address this gap, we launched a pilot initiative to develop the Chinese physical fitness standard for campus football players. Physical fitness is an essential ability for health and athletics and can be used as an objective criterion for identifying talent in youth football (Unnithan et al., 2012). Recently, the Chinese Football Association (CFA) released the first “Chinese Men’s Youth Player Athletic Ability Stage Evaluation Criteria (2021 Edition)” (Chinese Football Association, 2022), making it China’s sole youth-specific football physical fitness criteria. This CFA 2021 criteria, however, has two major limitations. First, it only applies to a narrow age range, primarily those between 13 and 16 years old. The campus football program encompasses a significantly broader age range, from primary school through college. Notably, one of the purposes of the campus football is to develop football reserve talent, with the most important target group consisting of young children in primary school, who are often under 12 years old in China. This highlights the need to address this age-related gap.

Second, the CFA 2021 criteria defines four levels of athletic ability: exceptional, good, passing, and failing. While this evaluation may suffice for general diagnostics of player development, the criteria could be improved by employing an advanced statistical technique. Specifically, the CFA 2021 criteria was formed by using the traditional quartile method (Chinese Football Association, 2022). It is not clear to us that the shape of the raw data used to create the CFA 2021 criteria. If data are non-Gaussian, the standard quartile method cannot be used to calculate z-scores. An advanced statistical approach, generalized additive models for location, scale, and shape (GAMLSS), has been presented by WHO as a solution for handling growth-related development (WHO MULTICENTRE GROWTH REFERENCE STUDY GROUP and de Onis, 2006). Briefly, GAMLSS enables customized statistical modeling with non-linear, non-parametric data and more comprehensive information from demographic data, and its application can be extended to other age-related development (Cole et al., 2009). Given that the State Council requires scientific criteria for the development of youth players, it is vital to explore alternate methods for establishing a national physical fitness standard for campus football players.

In this study, 9- to 11-year-old campus football players were sampled. In each age group, we evaluated four components of physical fitness, including flexibility, football-specific agility, speed, and lower body power. Using the GAMLSS method, this study set out to establish a framework for developing a centile-based Chinese physical fitness standard for campus football players.

## Methods

### Participants

The regulatory office of the Changsha Youth Sports and Medical Research Service Center approved this study’s protocol for the protection of human research participants. The collecting of data was assisted by trained researchers from Hunan Normal University. Before data collection, the parents of the children submitted written consent.

Volunteers in this study were recruited from the YFL Football Camps 2020 and 2022. As part of the campus football initiative, teams primarily from the Changsha region were invited to the football camp. All football camp participants were invited to participate in this research project. Because all players were male, only boys were selected. We collected data of 255 players aged 9, 221 players aged 10, and 289 players aged 11. The anthropometric characteristics of the participants are shown in Table 1.

**TABLE 1:** Summary of demographics.

Age (yrs)	Height (cm)			Weight (kg)			Body mass index (kg/m^2^)			Body fat (%)			Sitting height (cm)			Leg length (cm)		
	*M*	*S*	*n*	*M*	*S*	*n*	*M*	*S*	*n*	*M*	*S*	*n*	*M*	*S*	*n*	*M*	*S*	*n*
9	135.6	6.0	255	30.6	5.5	255	16.5	2.3	255	15.7	5.4	254	72	4	252	72	4	110
10	141.7	5.8	221	34.8	7.1	221	17.2	2.3	221	16.7	5.9	220	76	3	215	81	6	214
11	145.6	6.1	289	37.5	6.8	289	17.6	2.7	289	16.5	6.5	287	77	3	289	84	5	287

*M*, mean; *n*, sample size; *S*, standard deviation.

### Measurements

Data collection was conducted during the YFL Football Camps in Changsha. The method and sheet adhered to the “Manual for measuring the athletic ability of child and adolescent football players” developed by the CFA and China Institute of Sport Science (Chinese Football Association, 2020). Before the field testing, all testers received training and had their skills evaluated by a Changsha Youth Sports and Medical Research Service Center specialist. Before each day of testing, all testing equipment was calibrated in accordance with the manufacturer’s guide. Each testing procedure is described in detail below. Due to the testing of a large number of child players in a condensed timeframe, the testing order was determined based on the teams’ convenience.

Height and weight: participants removed their footwear, dressed lightly, stood upright on the measuring instrument (Zhikangyuan Health Technology Co., China), and looked straight ahead. The tester then recorded participants’ height and weight.

Body fat percentage: on the upper arm, scapulae, abdomen, and thigh, body fat was measured using a skinfold caliper (Zhikangyuan Health Technology Co., China). Participants stood naturally while the tester used the thumb, index finger, and middle finger of the left hand to pinch and lift the skin and subcutaneous tissues of the tested area. The skinfold caliper was then used to measure the thickness of the skinfold 1 cm below the pinch point. Three measurements were made in total, with the average value or the same value taken twice. The Nagamine and Suzuki equation was used to calculate body density (Nagamine and Suzuki, 1964), followed by the Brožek equation to determine the body fat percentage (Brožek et al., 1963).

Sitting height: participants sat on a measuring chair (adult and child height and sitting height meter, Saiao Industrial Co., China), straightened the body, maintained a vertical line between the left and right shoulder blades, and did not adhere to the chair’s back. The tester determined the sitting height as the vertical length from the top of the head to the ischial tubercle.

Leg length: the distance between the participant’s right anterior superior iliac spine and the ground was determined with a measuring tape.

Sit and reach test: participants sat on a mat with both feet against the test meter (Aopi, China), knees extended, and slowly flexed the body forward, using the tips of the middle fingers on both hands to push the equipment slide until the maximum range is reached. If a sudden force occurred or both knees were bent, a retest was conducted.

*t* test: participants began running forward from the beginning point located at A, arrived at point B, slide to the left to move to point C, slide to the right to move to point D, slide to the left to travel back to point B, and then ran backwards to the starting point located at A. Participants were required to complete two separate tests, and the faster test was recorded (Freelap, Switzerland).

30 m run test: participants began in a standing position and ran 30 m as quickly as possible. Participants were required to complete two separate tests, and the faster test was recorded (Freelap, Switzerland).

Vertical jump test: participants stood on the vertical jumping mat (OptoJump, Italy), stood straight and squat rapidly, then jumped vertically upwards (hands can swing) and landed in the same place.

### Modeling and statistics

The centile estimation of physical fitness standard was performed using open source *RStudio* version 2022.07.1 + 554 and modelling in *gamlss* version 5.4-3. In accordance with Cole’s original Lambda-Mu-Sigma method (Cole, 1988), the recommended first step of centile estimation is to chooses an appropriate power transformation of age. In this study, this step was omitted. The power transformation of age is necessary for specific age ranges associated with exponential child growth, such as infant and toddler before age 5 (WHO MULTICENTRE GROWTH REFERENCE STUDY GROUP and de Onis, 2006), or for a broad age range that could confound the child and adult trends (Cole et al., 2009). In our limited-age-range test, neither condition has an effect on the overall modeling fit.

Based on the preliminary check of data distribution, we fitted different GAMLSS families of distributions to each physical fitness test. For the *t* test, 30 m run test, and vertical jump test, we compared the Box-Cox Cole and Green (Cole and Green, 1992), Box-Cox power exponential (Rigby and Stasinopoulos, 2004), and Box-Cox *t* (Rigby and Stasinopoulos, 2006). For the sit and reach test, we compared the skew *t* type 3 (Fernández and Steel, 1998), skew exponential power type 3 (Fernández et al., 1995), and original Sinh-Arcsinh (Jones and Pewsey, 2009). Each parameter (mu, sigma, nu, tau) of a distribution was fitted locally using the P-spline smoothing function based on the local maximum likelihood method and coupled with default link functions in *gamlss*. Following these steps, the Schwarz Bayesian criterion was used to select the best-fitting model (Voudouris et al., 2012), which corresponds to the smallest Schwarz Bayesian criterion value. The worm plot (Buuren and Fredriks, 2001) was used to confirm the global goodness of fit. In addition, we used the Shapiro–Wilk test to validate the normality of the residuals. As the final step, the centile estimation of a suitable GAMLSS model was computed for each physical fitness test.

Using the information from this data set, we also conducted a factor analysis to determine the impact of childhood overweight and obesity on physical fitness testing. The body mass index was used to divide each age group’s participants into normal weight and overweight/obese categories. Specifically, overweight/obesity is defined as a body mass index greater than 18.9, 19.6, and 20.3 kg/m^2^ for Chinese boys aged 9, 10, and 11 years old, respectively (Group of China Obesity Task Force, 2004). Welch’s *t*-test was performed using the GraphPad Prism version 9.0.0 and *p* value of less than 0.05 indicates statistical significance.

## Results

**FIGURE 1:**
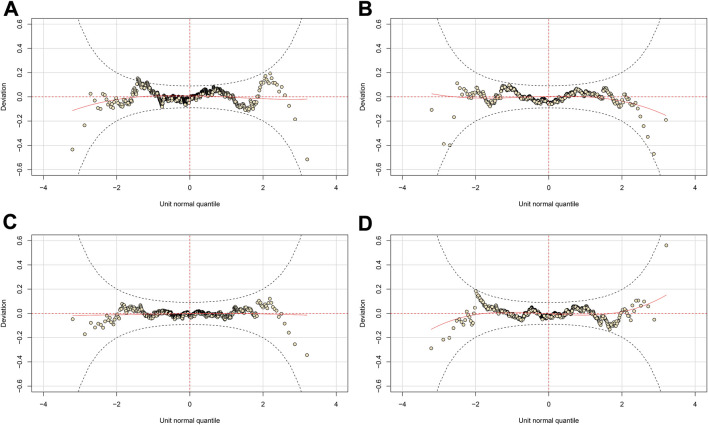
Worm plots of fitted GAMLSS model. **(A)** Sit and reach test; **(B)** *t* test; **(C)** 30 m run test; **(D)** vertical jump test.

When the physical fitness data were fitted with a normal distribution in *gamlss*, the normalized quantile residuals indicated that none of the data adhere to a normal distribution, hence justifying the subsequent modeling process. Except for the vertical jump test, all other physical fitness tests require specific GAMLSS distributions with a kurtosis-controlling parameter. Table 2 summarizes the selected distributions to fit the model. After transformation, all fitted models have a mean close to 0, a variance close to 1, a coefficient of skewness close to 0 and a coefficient of kurtosis close to 3. Using worm plots (Figure 1), the quantile residuals of each fitted model were visually examined. Given that the majority of points in worm plots fall within the two elliptic curves, this indicates that the modelling is acceptable. This is cross-validated using the Shapiro-Wilk normality test. The *p* values were 0.1274 for the sit and reach test, 0.2851 for the *t* test, 0.902 for the 30 m run test, and 0.5955 for the vertical jump test. Following these diagnostic procedures, centile estimation for physical fitness tests between the ages of 9 and 11 was computed and given in Table 3.

**TABLE 2:** Summary of fitted GAMLSS model parameters.

Test	*n*	Distribution	Smoother	*edf*				SBC
				*μ*	*σ*	*ν*	*τ*	
Sit and reach	748	SHASHo	pb()	2.00	2.00	2.91	2.62	4,724.648
T	731	BCPE	pb()	2.88	2.00	2.99	2.96	1898.066
30 m run	730	BCPE	pb()	2.00	2.00	2.00	2.86	804.6495
Vertical jump	760	BCCG	pb()	2.71	2.92	2.56	N/A	4,537.898

BCCG, Box-Cox Cole and Green; BCPE, Box-Cox power exponential; *edf*, effective degrees of freedom; *n*, the number of observations in the fit; N/A, not applicable; *pb*(), P-splines smoothing function in *gamlss*; SBC, schwarz bayesian criterion; SHASHo, original Sinh-Arcsinh; *μ,* mu, *σ,* sigma, *ν,* nu, *τ,* tau.

**TABLE 3:** Physical fitness standard for campus football players aged 9 to 11.

Test	Age (yrs)	*n*	Percentiles										
			1	3	5	15	25	50	75	85	95	97	99
Sit and reach (cm)	9	253	−8.4	−4.9	−3.2	0.9	3.0	6.5	10.5	13.1	18.4	20.7	25.3
	10	221	−10.8	−6.9	−4.9	−0.3	2.1	5.9	9.2	11.0	14.4	15.8	18.6
	11	274	−4.7	−2.3	−1.0	2.1	3.8	6.9	9.8	11.3	14.1	15.2	17.3
T (s)	9	251	16.55	15.88	15.51	14.50	13.85	12.65	11.77	11.47	11.12	11.03	10.88
	10	212	15.30	14.11	13.61	12.67	12.30	11.84	11.48	11.31	11.07	10.98	10.83
	11	268	14.53	13.53	13.14	12.38	12.03	11.52	11.12	10.93	10.65	10.55	10.37
30 m run (s)	9	251	7.55	7.04	6.82	6.35	6.12	5.78	5.49	5.34	5.10	5.01	4.85
	10	211	7.35	6.72	6.48	6.04	5.85	5.60	5.39	5.28	5.08	5.00	4.86
	11	268	6.66	6.30	6.15	5.82	5.67	5.42	5.23	5.14	5.02	4.97	4.89
Vertical jump (cm)	9	252	14	16	17	20	22	25	28	29	32	33	35
	10	219	18	20	21	23	24	27	30	31	34	35	37
	11	289	16	18	20	23	25	28	32	34	37	38	40

**FIGURE 2:**
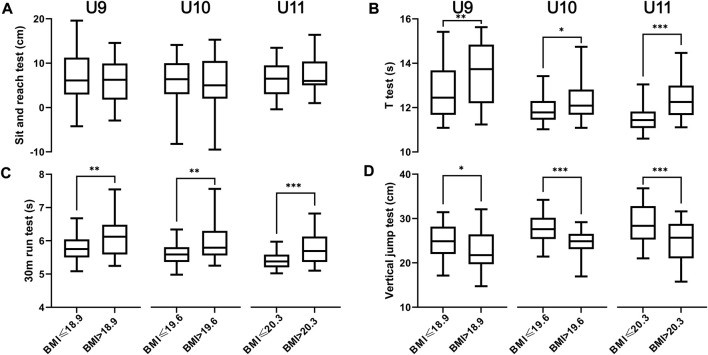
Box plots (presented as medium with 95% confidence interval) show the impact of body mass index on physical fitness testing in campus football players aged 9 to 11. **(A)** Sit and reach test; **(B)** *t* test; **(C)** 30 m run test; **(D)** vertical jump test. * denotes *p* < 0.05; ** denotes *p* < 0.01; *** denotes *p* < 0.001.

Figure 2 depicts the influence of BMI on the physical fitness of campus football players aged 9 to 11. Except for the sit and reach test, overweight and obese children performed significantly worse on agility, speed, and lower body power measures specific to football.

## Discussion

In this study, we assessed a total 765 children participating in a campus football summer camp. While the training background of this population is unknown, our 10 year old age group (see the 50th percentile in Table 3) performed comparably to the same age group from English lower-league development programmes (Lovell et al., 2015) based on the *t* test and vertical jump test. Similarly, the performance of the 10 and 11-year-olds in our study on the 30 m run test was comparable to that of elite youth players of the same age from Belgium (Rommers et al., 2019). Although physical fitness is not the main driver of football competence, our participants represent a valid sample of campus football players near the Changsha region.

This is the first modeling study in China to establish the physical fitness standard for campus football. Using the state of art GAMLSS method, we are able to transform the highly skew and kurtotic data into a distribution bias-free pattern and create the first comprehensive rating standard with 0–100 percentiles. The benefit of this novel standard is evident when compared to the most recent CFA 2021 criteria, which only permit rating physical abilities on a four-level range. In application, our standard offers a wide variety of uses. For diagnostic purpose such as maturity levels of player development (Rommers et al., 2019), the bottom 15th percentiles of our standard can be utilized to identify late-maturing players and give objective reference for intervention measures. Likewise, for school enrollment and college admission based on football ability, the top 15th percentiles of our standard can serve as an objective scoring system for talent identification. The implementations of such percentile-based standard could usher in China’s most rigorous and scientific campus football player assessment standard, which would be consistent with the Ministry of Education’s most recent key task list (Ministry of Education of the People’s Republic of China, 2022).

In China, the establishment of youth player athletic ability criteria is still in progress (Chinese Football Association, 2022). Establishing a population standard is not only expensive and labor-intensive, but also requires meticulous planning to ensure that the outcomes are based on the scientific rigor method. Based on this pilot study, we advise the Ministry of Education and CFA consider the following three recommendations for developing the future national physical fitness standard for campus football. The first consideration in planning a centile research is determining the ideal sample size to measure, which must take into account both the accuracy of estimation and the financial feasibility of the project. While the global goodness of fit is considered adequate for this technical pilot study, the small sample size clearly affects the fit at the extremes of the age. For example, the worm plot for the vertical jump test displays an S-shaped curve, indicating that the few and outlier samples in the two tail ends diminish the testing power of the model. A smoothed GAMLSS model is supported not just by data at specific ages, but also by data at adjacent ages, a process that “borrows strength” throughout the age span (Rice, 2004). Cole has presented theoretical foundations for determining the optimal sample size for GAMLSS based centile research and suggested 7,000–25,000 participants per sex for the reference of the 0–20-year-old population (Cole, 2021). Regarding this subject, there is one China-specific issue and one China-specific advantage. The issue is that the physical activity (Shi et al., 2006), body mass index (Zhang and Wang, 2012), and physical education (Gui and Yu, 2021) of Chinese children and adolescents vary greatly by socioeconomic location, which is anticipated to correlate with regional differences in the physical fitness of campus football players. The advantage is that the Chinese government is dedicated to assist the full growth of the campus football environment, including the management of big data. In the most recent key task list (Ministry of Education of the People’s Republic of China, 2022), it is stated explicitly that “Improve the level of information technology. Establish electronic files of campus football players...Through comprehensive digitization of...data archiving, result tracing, and process supervision are realized to provide a basis for decision making for campus soccer development and help campus football work develop with high quality”. Given the regional variation in player physical fitness, the minimum sample size for the development of a national physical fitness standard should consist of 2,000 participants (Cole, 2021) from each province of China, with all regional samples then being combined for a total of 62,000 participants. Our recommendation is to take use of the era of information technology and build the most precise national physical fitness standard for campus football by utilizing all archived player statistics. In keeping with the broader Chinese football reform (The State Council of the People’s Republic of China, 2015), Chinese social enterprises that adhere to the law and ethics of big data management should be encouraged to take part in the formulation of the standard.

The second recommendation comes from our experience in sports statistics. There is known to be a relative age effect on the biological maturation of young football players, which not only results in anthropometric variances (e.g., height, leg length) (Gil et al., 2014) but also could lead to a systematic bias during talent development in favor of players born early in the year and early maturers (Williams, 2010). In terms of relative age effect on physical fitness, Gil and others discovered that nine- to 10-year-old male football players born in the early months of the year performed better on the 30 m run test, agility test, and overall performance score than their peers of the same age born later in the year (Gil et al., 2014). Therefore, while an age difference of less than 12 months may not be relevant for evaluating children’s performance in basic physical education curriculum, it may be significant in high performance sports. Fortunately, this bias could be easily resolved by recording data on age and testing time. Future nationwide testing should include players’ birth year and month on the data sheet and in online archives, along with the testing year and month. When modeling centile estimation, researchers could classify players’ testing age, resulting in a more precise physical fitness standard.

The third recommendation is related to field procedure, which is a summary of our experience from this study. The CFA manual (Chinese Football Association, 2020) (effective) provides a clear and scientific guideline for testing young football players. However, this lacks a necessary clarification for researchers developing national physical fitness standard. In order to standardize the field procedure, CFA should amend its manual to include the particular testing order and the specific testing time gap between each test battery, as well as a practical time range allowance. This refinement would further improve the rigorousness of a national physical fitness standard.

Finally, the effect of child and adolescent obesity on health and physical fitness has been extensively documented in the literature, and the age range examined in this study is well known to be crucial for the athletic development in young athletes (Bergeron et al., 2015) as well as motor competence in the general population (Haga, 2008). Rather, our results should be broadly considered in the context of the national fitness program. Campus football is intended to strengthen China’s football competency on the international stage, but more importantly, it is part of the national policy to improve the health of the entire Chinese population (The State Council of the People’s Republic of China, 2021), which is a nation’s true core competency. The Chinese government believes that it is vital to take real steps to combat the increasing number of overweight and obese children (Wang et al., 2021), and that campus football is an ideal type of physical activity for this purpose. Our findings provide the first valid benchmark for future longitudinal research comparing the overall effectiveness of campus football in Changsha and beyond.

In conclusion, this is the first modeling study that lays the foundation for the creation of national physical fitness standard for campus football. The significance of our findings lies in their potential to facilitate the development of a comprehensive, scientific, and contemporary national fitness program.

## Data Availability

The data that support the findings of this study are available from the corresponding authors upon reasonable request. Requests to access the datasets should be directed to Wei Deng, dengwei@gduf.edu.cn; Zeyong Liu, lzy1002679102@163.com.
